# New Graduate Nurses Navigating Entry to Practice in the Covid-19 Pandemic

**DOI:** 10.1177/08445621221150946

**Published:** 2023-01-12

**Authors:** Kim McMillan, Chaman Akoo, Ashley Catigbe-Cates

**Affiliations:** 1 School of Nursing, University of Ottawa, Ottawa, ON, Canada

**Keywords:** Covid-19, new graduate nurse, transition, nursing, education, socio-political knowledge

## Abstract

**Background:**

The Covid-19 pandemic has significantly impacted organizational life for nurses, with known physical and psychological impacts. New graduate nurses are a subset of nurses with unique needs and challenges as they transition into their registered nurse roles. However, this subset of nurses has yet to be explored in the context of the Covid-19 pandemic.

**Purpose:**

To explore the experiences of new graduate nurses entering the profession in Ontario, Canada, during the Covid-19 pandemic approximately one year after entering the profession.

**Methods:**

Thorne's interpretive description method was utilized.

**Findings:**

All participants identified as completing second entry nursing programs, offering a unique perspective on new graduate nurse transition. Four themes emerged in the data: ‘*Virtual Didn’t Cut It,’ ‘Go Where You Know,’ ‘Picking Up the Pieces,’ and ‘Learning When to Say No and Let Go.’* Participants felt ill prepared to enter the profession and were cognizant of the various challenges facing the nursing profession, and how these pre-existing challenges were exacerbated by the pandemic. They acknowledged the need to protect themselves against burnout and poor mental health, and as such, made calculated early career decisions – demonstrating strong socio-political knowing. Half of the participants had already left their first nursing job; citing unmet orientation, mental health, and wellbeing needs. However, all participants were steadfast in remaining in the nursing profession.

**Conclusions:**

Second entry new graduate nurses remain a unique subset of nurses that require more scholarly attention as their transition experiences may differ from the traditional trajectory of new graduate nurses.

## Background and purpose

Covid-19 was declared a global pandemic by the World Health Organization [WHO] on March 11, 2020. Canada saw its first confirmed case January 27th, 2020, with Ontario being one of the hardest hit provinces, recording some of the highest numbers of cases in the country (Government of Canada, 2022). The ensuing public health measures and restrictions impacted graduating nursing classes of 2020, adding complexity and disruptions to their schooling experience and subsequent entry to practice (Organization of Nurse Leaders [ONL], 2021). Clinical placements were suddenly halted during the 2020 winter term (Monforte-Royo & Fuster, 2020), which for many baccalaureate programs, is when fourth year nursing students are consolidating their clinical knowledge in intensive clinical rotations before entering the profession (commonly referred to as “consolidation”). Some consolidations slowly resumed, while others did not, instead providing students with virtually simulated formats of knowledge consolidation. Students reported significant uncertainty in response to these disruptions (Naylor et al., 2021). The experiences of nursing students during the Covid-19 pandemic are actively being captured in a growing body of literature, notably highlighting challenging transitions to alternative ways of learning (e-learning) (Daynes, 2020; Hodges et al., 2021; Swift et al., 2020), interruptions to clinical placement experiences (Ulenaers et al., 2021), and the psychological stress these disruptions had on student nurses (Aldridge & McQuagge, 2021; Espin et al., 2021).

There is also a growing body of literature illuminating the complexity of new graduate nurse transition during the Covid-19 pandemic. Collectively the findings highlight experiences of fear, uncertainty and self-doubt (Aukerman et al., 2022; García–Martín et al., 2021; Kovanci & Atlı Özbaş, 2022) alongside negative impacts on psychological health (Katsuta et al., 2021; Nayor et al., 2021), while other findings reveal themes of resilience and commitment to the profession of nursing (Casey et al., 2021; Nayor et al., 2021; Sessions et al., 2021). Some of these emerging experiences have also been reflected in past endemics, however the longevity and magnitude of the Covid-19 pandemic may serve to mediate experiences in ways that may not parallel these past experiences.

The literature pertaining to new graduate nurses’ transition during past endemics including SARS (2003), H1N1 (2013), MERS-CoV (2015), and Ebola (2018) is sparse and conflicting. Some studies suggest the psychological impacts on new graduate nurses was significant, and included: anxiety, depression, fear, sleep disturbances, loneliness, and post-traumatic stress disorder (Sun et al., 2020; Wong et al., 2004). While others suggest the unique new graduate experiences during such times (specifically SARS) fostered a strong sense of professional identity and resiliency (Heung et al., 2005). Furthermore, previous endemic research suggests new graduate nurses felt an intense ethical commitment to ‘do their bit’ and ‘make a difference’ (Slettmyr et al., 2019). As such, they struggled to navigate the delicate balancing act of protecting and caring for the self, and protecting and caring for others, which are often in a state of dissonance in nursing (Chen et al., 2005; Swift et al., 2020). Similarly, emerging Covid-19 literature suggests that student nurses and new graduate nurses alike, felt the “call to duty” (p. 1), which was not without risk and personal sacrifice (Espin et al., 2021). Such dissonance could be further compounded by the ‘hero’ narrative popular media outlets and government officials have attached to nurses on the front lines of the Covid-19 response. For some practicing nurses, the hero narrative has fostered a perception that “appreciation is a reward given only to those who put themselves in danger” (Swift et al., 2020, p. 3112). We do not yet know how new graduate nurses are navigating these complex narratives in the context of Covid-19, and what their subsequent experiences of transition are.

Furthermore, few guidelines on how to support new graduate nurses specifically during Covid-19 have been produced, the most notable and substantive from the Organization of Nurse Leaders [ONL] (2021). The 2021 report suggests a need to rebuild preceptor capacity and create community amongst new graduate nurses as social distancing restrictions put them at greater risk for isolation. Additional supports should include frequent debriefing, storytelling, and reflecting within the new graduate nurse-preceptor dyad (ONL, 2020). These guidelines are supported elsewhere in the emerging Covid-19 literature (Russel & Juliff, 2021). These proposed guidelines build upon what is already known about the complex phenomena of transition experienced by new graduate nurses (Duchscher, 2008; 2009; Dyess & Sherman, 2009).

During the transition period, which is noted to be anywhere between 12 months (Duchscher, 2008) and three years (Monagle et al., 2018), new graduate nurses experience what has been described as a problematic developmental lag when entering the workforce, stemming from differing expectations of previous educational stakeholders and current workplace stakeholders (Ortiz, 2016; Rush et al., 2019; Wolff et al., 2010). The transition period for new graduate nurses is challenging, demanding, and has even been described in the literature as traumatic (Dwyer & Hunter Revell, 2016; Edwards et al., 2015; Powers, Herron & Pagel, 2019).

In order to successfully transition into confident, independent practitioners, new graduate nurses require mentorship and ongoing support in developing clinical competencies (College of Registered Nurses of British Columbia [CRNBC], 2009; Del Bueno, 2005; Powers et al., 2019), the ways in which these needs are met are multi-pronged, occurring across the trajectory of the transition period. An (updated) integrative review by Rush and colleagues (2019) suggests the length of formal transition programs vary between less than one month to 36 months, noting informal supports should continue past this timeframe. Strong and consistent support throughout the transition period results in benefits at the individual and institutional levels. Individual new graduate nurses report higher levels of job satisfaction, confidence and clinical leadership, enhanced critical thinking skills, social integration, and less emotional exhaustion turnover intent, and reality shock compared with counterparts who did not have formal transition supports (Chappell & Richards, 2015; Edwards et al., 2015; Kramer et al., 2013; Laschinger & Grau, 2012;). Consequently, institutions benefit from significant cost savings attributed to higher rates of retention (Bradley, 2017; Cubit & Ryan, 2011; Fox, 2010).

Historically, investments in new graduate transition programs have provided long-term cost benefits (Rush et al., 2019) and enhanced new graduate wellbeing. There is currently an urgent need for robust empirical evidence on how best to structure and provide new graduate nurse transition programs that can still assist with retention and building competency during incredibly challenging times, such as a global pandemic. Robust investments in new graduate nurses work to support the human health resource drains and the rapidly increasing rates of nurse attrition seen in Canada (Nurses and Nurse Practitioners of British Columbia [NNPBC], 2021; Registered Nurses Association of Ontario [RNAO], 2021), however such investments may be challenging during increasingly austere fiscal measures noted in healthcare.

What is important to note when further contextualizing what is known about new graduate nurse transition is that there are varying types of new graduate nurses that may not be consistently reflected in the new graduate nurse transition literature. For example, there are new graduate nurses who complete non-traditional nursing degrees which include second-entry programs (also be referred to as *accelerated, fast-track, compressed,* and *advanced entry)* (Canadian Association of Schools of Nursing [CASN], 2019). There is literature that suggests these second entry nursing students are unique, for example, it is suggested that as practicing nurses they exhibit greater maturity and motivation compared to their traditional-stream counterparts (Codier et al., 2015; Penprase, 2012). This unique cohort of nurses have also garnered recognition from employers due to their outstanding emotional intelligence, maturity, and ambition (Oermann et al., 2010; Shatto et al., 2016). Second entry nurses are also said to be highly aware of their needs and are able to better voice those needs in the workplace when compared to nurses graduating from traditional nursing programs (Brandt et al., 2017). It is further suggested second entry nurses “are likely to have more experience setting and achieving goals, overcoming challenges, engaging in mastery experiences, receiving feedback, and learning vicariously through role models” (Read & Laschinger 2017, p.54). Entry to practice literature does not make this critical distinction, so it is unclear when these distinctions occur in the career trajectory, and notably in the current pandemic context – if second entry new graduate nurses transition to practice differed in any way from traditional new graduate nurses.

It is important to understand the impact of the Covid-19 pandemic for new graduate nurses (from both traditional and non-traditional programs), as the first year of clinical nursing practice strongly influences a nurses’ decision to stay in the profession, or leave (Morgan, 2017). This attrition further exacerbates a pre-existing nursing shortage (The McGill Nursing Collaborative for Education and Innovation in Patient- and Family-Centered Care, 2019). The rate at which new graduate nurses leave the profession within their first two to five years of practice is higher than all other cohorts of more senior nurses (mid-career, mid-to-late career, late-career) in many areas of the world, including Canada (Fiester, 2012; Kovner et al., 2014; The McGill Nursing Collaborative for Education and Innovation in Patient- and Family-Centered Care, 2019; Trepanier et al., 2012). This data is troubling, as new graduate nurses are the main supply of annual nursing labor force increases (Canadian Institute of Health Information [CIHI], 2019), and they are a vital health human resource (Regan et al., 2017). The average age of practicing Registered Nurses [RNs] in Canada is 44 years, with approximately 20% of Canadian RNs being 55 and older (CIHI, 2021), noting that 85% of Canadian RNs retire before the eligibility age of 65 (Hewko et al., 2019). As such, it is imperative to keep as many new graduate nurses as possible in the profession to ease the current nursing shortages which are at an all-time high globally (International Council of Nurses, 2022), with some countries reporting skyrocketing increases in nursing job vacancies. For example, in Canada, when compared to first quarter 2020 data, Registered Nurse (RN) vacancies have risen by 78% in the first quarter of 2022 (Statistics Canada, 2022). Better understanding the unique experiences, and needs, of Covid-19 new graduate nurses can serve as a starting point to bolster retention efforts for this invaluable group of newly registered nurses.

The purpose of this study was to gain knowledge about how new graduate nurses’ transitions have been impacted by the Covid-19 pandemic. The impetus for this inquiry was inspired by the Principal Investigator's (PI) observations of the many anxieties and fears expressed by their 2020 cohort of fourth-year nursing students, particularly when consolidation experiences were cancelled and the pandemic intensified in the province of Ontario. The research question that guided this study was: 1) What are the experiences of new graduate nurses transitioning into their roles as registered nurses in the midst of the Covid-19 pandemic in Ontario, Canada?

## Methods and procedures

The social ecological model and relational ethics theoretically framed this study. The social ecological model proposes that individual experiences are heavily influenced by their surrounding environment (Crosby et al., 2013; McLeroy et al., 1988; Stokols, 1996), highlighting that experience is not merely the result of individual characteristics (Drew et al., 2016). In this model, the environment consists of five interrelated elements: intrapersonal (individual characteristics), interpersonal (the ways in which an individual engages with others), organizational (processes embedded in organizations), community/sociocultural factors (values and beliefs of wider society), and public policy (Drew et al., 2016; McLeroy et al., 1988). The level of compatibility between a person and their environment strongly shapes lived experience (Stokols, 1996). For the purpose of this study, all aforementioned environmental elements were considered to better understand new graduate nurse transition during the Covid-19 pandemic. Previous documented use of this framework in the nursing literature, specific to that of new graduate transition (Dwyer & Hunter Revell, 2016), focused on specific elements of the environment, however, in light of the sweeping impacts of the Covid-19 pandemic, at all levels of environments - both personally and professionally, all five environments were used to frame this study. The social ecological model is a relevant framework to aid in understanding how new graduate nurses’ current environments may be interacting and shaping their transition experience during the Covid-19 pandemic.

The pandemic has also brought to light highly ethically charged narratives, which have mediated nursing work (Branicki, 2020; McMillan et al., 2021; Morley et al., 2020; Sperling, 2020; Turale et al., 2020). Therefore, ethical framing of this study was also deemed important. Because the social ecological model focuses on the intricate relationships between all environmental elements, a relational ethical framing worked to compliment the framework by making visible the relational practices occurring as elements of one's environment interrelate. Relational ethics is the framing of relationships as highly ethical encounters and is described as an “action ethic” that recognizes the interdependence between individuals and their surrounding environments (Bergum & Dossetor, 2005). It acknowledges the importance of the broader environment in shaping individual experiences and subsequent action. It helps further understand day-to-day moments that occur between people and the elements of their environments (Deschenes & Kunyk, 2019). Furthermore, relational ethics focuses on relational spaces and the quality of relationships, with a keen interest in better understanding the relationships that allow for the creation of mutual trust, engagement, and embodied knowing (Bergum, 2004), making it an important lens to interpret the difficult and challenging new graduate nurse transition phenomena.

The study design was interpretive description (Thorne, 2016) – an approach designed to provide practice-relevant knowledge for clinical nursing. A qualitative approach was used to develop an in-depth understanding based on new graduate nurses’ experiences of transition. New graduate nurses from any healthcare setting in Ontario who graduated in the Spring 2020 cohort of nursing degree programmes were invited to participate in a single interview on their transition during the Covid-19 pandemic. In order to participate in this project, participants had to be able to communicate in English and have access to a working internet connection, as all interviews were conducted virtually over a secure zoom meeting room. Recruitment occurred via social media and through personal, academic, and clinical nursing networks across the province of Ontario. Early on in data collection it was noted that the first three participants were second-entry new graduate nurses, as opposed to traditional (4 year) graduates. A return to the literature noted differences in these two populations of nurses, as discussed in the background section. It was also noted that this unique subset of nurses; notably when they are experiencing new graduate transition, had yet to be captured in the emerging Covid-19 literature. The decision was thus made to focus data collection solely on second-entry new graduate nurses. Snowball sampling was also used to recruit this subset of new graduate nurses – participants were encouraged to share recruitment material (electronic recruitment poster) with anyone they thought may want to participate. Data collection occurred between June and July of 2021. Data was collected by the PI, who has six years of experience teaching this subset of nursing students, as well as clinical experience precepting them as new graduates. All interviews were also supported by one of two Research Assistants (RAs) – one at the graduate level, and one at the undergraduate level – both of whom have or are currently completing traditional nursing programs which also include(d) second-entry students in the latter years of study (year 3 and 4).

Because of the time-sensitive nature of the inquiry (to rapidly produce knowledge that can be available to support new graduates entering the profession during the pandemic) interviews were analysed directly with no verbatim transcription. Using an inductive approach, recordings were viewed multiple times by the Principal Investigator and Research Assistants. Reflections on the data as it related to the aims of the research and theoretical underpinnings were captured and notes of salient points and preliminary insights were made. In depth analysis of each participant's interview were then performed. The ways in which participants engaged with, and navigated, the complexity of the various environments outlined by the social ecological model (Drew et al., 2016; McLeroy et al., 1988) structured the data analysis process. Analysis was further nuanced by examining the quality of relationships in those various environments and the ways in which participant experiences embodied relational practice. Subsequent codes specific to segments of discourse that provide partial answers to the research question were applied. An interpretive synthesis of each participant's interview was then written, which were then read together to achieve an analysis across participants narratives. Weekly meetings with the research team throughout the analysis process supported ongoing immersion in the data.

The study was developed with rigorous attention to the Tri-Council Policy Statement: Ethical Conduct for Research Involving Humans (Government of Canada, 2018). Ethics approval was granted for this study. There was a risk of negative social repercussions for participants should their comments made in the context of this study become known, either to the participant's employer, or within the professional community. This risk was low, as the researcher kept all data collected strictly confidential. All data was anonymized and remains securely kept. No data identifying where participants work was collected. The participant's identity was not known to anyone outside of the research team which consisted of the Principal Investigator and two Research Assistants. It was up to participants whether they chose to disclose to others that they participated in this study. As with any qualitative interview with nurses about their work, this project involved discussions of a sensitive nature and therefore, there was always the possibility that participants could become upset by these conversations. It is important to point out, however, that in the PI's history of conducting interviews of this nature with RNs, the impression has been that the benefit of having the opportunity to voice their perspectives and experiences far outweighs any risk of temporary emotional discomfort. Participants were provided an opportunity to share their concerns and experiences on this important topic, which in itself may prove to be beneficial. Also, participants may potentially feel a sense of connection to the broader new graduate nursing community by contributing to this project, which may be particularly helpful as data was collected during ongoing social distancing.

## Results

The sample size was eight new graduate nurses that graduated in the Spring of 2020. Interviews were conducted over zoom, lasting between 50 and 70 min. All participants were second entry program graduates from multiple Ontario postsecondary institutions. Data saturation occurred at participant eight. Five of the participants had their final student consolidation experiences fully cancelled; three had consolidation cut short. At the time of data collection all participants held at least two nursing jobs co-concurrently, while over half had already left a nursing job in an acute care hospital. Participants worked in a variety of settings including acute care (urban and rural; adults and pediatrics) and public health and community care. All participants stated they were working full time hours or above, but that these cumulative hours came from more than one nursing job. All participants held two jobs, one participant held three. Two participants held full time hours at one of their jobs (plus another part time job), whereas the remaining were part time employees at all jobs. Four salient themes emerged from the data; (1) Virtual Didn’t Cut It; (2) Go Where You Know; (3) Picking Up the Pieces and (4) Knowing When to Say No and Let Go. Each will be discussed with more depth and granularity below.

### Virtual didn’t cut It

This thematic finding attends to the intrapersonal (individual characteristics), interpersonal (the ways in which an individual engages with others) and organizational (processes embedded in organizations) environments within the social ecological model, which theoretically guided the study and drove data analysis. Traditional consolidation was described as the capstone milestone that “ties it all together” and serves as the gatekeeper to successfully transitioning into new graduate nursing. Participants described consolidation as a time when they could truly embody the nursing process, develop time management skills, inter- and intrapersonal skills, and navigate complex political and power structures. Contrastingly, virtual consolidation was described as “reductive and simplistic,” one participant decidedly called it a “joke,” another highlighted that, “it absolutely did not capture the complex dynamics [of nursing work environments].” Time management skills went unmet because, as one participant stated, “in virtual clinical it was step one, step two…but in a real environment there's five things going on at once, and I need to prioritize.” Another participant highlighted that virtual consolidation did not capture the complex dynamics of the work environment, stating:

What virtual consolidation didn’t teach us was, what does a good work environment look like when working as a nurse; what is a safe staff-patient ratio? What are the attitudes of colleagues and how do you respond to those…there's so many egos…so as a new grad, how do you navigate working with an older nurse who eats their young…or how do I ask a difficult resident [doctor] to come assess my patient?

Participants felt the absence of a traditional consolidation left them ill-prepared, which took a significant emotional toll on them. As one participant noted, “It [nursing as a new grad] was terrifying, I was so ill-prepared without consolidation,” while another participant reflected “without having that capstone of consolidation I doubt myself a lot…even still [one year in].”

Participants also felt betrayed by their schools and educators. Multiple participants said they were told by their educational institutions and educators that it would be ok that they didn’t have a traditional consolidation, that their respective workplaces would support building capacity and confidence as they navigated entry into their professional roles - participants voiced that this had absolutely not been the case. Orientations had not been adapted for these new graduates and in some instances, orientations were cut short to mitigate significant staffing issues or because orientation funding had been redirected to alternative institutional activities and priorities. One participant also described having to preceptor a fourth year consolidation nursing student from the subsequent 2021 cohort, because there was simply no other individual on their unit that had the capacity to support the new nurse. Cumulatively these experiences created a significant dissonance in what new graduate nurses thought they would experience entering the profession, versus their lived realities. This was acutely evident in the aforementioned participant, who questioned how they could be positioned to mentor and support a consolidation experience for a student, when they themselves had not had this experience.

### Go where you know

This thematic finding also attends to the intrapersonal (individual characteristics), interpersonal (the ways in which an individual engages with others) and organizational (processes embedded in organizations) environments within the social ecological model. As a result of feeling ill prepared in the absence of consolidation and fearing the unknown amidst a global pandemic, participants made very calculated decisions about where they started their nursing careers. Participants deliberately chose to start their careers in places they previously had positive clinical experiences, with one participant stating:

“It's more important to feel comfortable with the culture of the unit than the [patient] population…that's why I started where I started…I went to a unit I knew: I knew the culture, I knew management, I knew staff”

Their rationale for making these calculated decisions is captured in the following participant quote: “I had a good sense of how things were, so it provided an idea of what I would encounter [as an RN].” Another participant explained that “…people [new graduate nurses] went to where they knew, where they knew the safety standards [to protect them against Covid-19], they knew the organizational funding for nursing - they sought out places they knew would protect them a bit more.” Participants chose to work in environments they knew would support them, and where a culture of inquiry was supported, and ultimately expected. All participants spoke about the need to be able to safely ask questions, especially in light of their insecurities as it pertained to the absence of consolidation. If they had experienced a positive work culture somewhere as a student, they gravitated to those units as new graduates, even if these units were not the specialty area they had aspired to work in. At this early point in their career amidst a global pandemic these new graduate nurses, “needed to know what I was walking into.”

Additionally, as much as the Covid-19 pandemic created apprehension about what was to come for these new graduate nurses, (which will be highlighted in the next theme), it also gave them profound insight into nursing working conditions, which had become highly publicized during the pandemic. As one participant illustrated, “The veil was lifted.” The veil lifting helped participants choose where to work once they graduated, and for some, changed their minds about working in areas they once thought they’d like to, as one participant elaborated: “I always wanted to work in Long Term Care, but I won’t now – the support structures for nurses are non-existent.” Although these new graduates had the insight to make such calculated decisions, they were still entering a highly fractured system, which left them feeling as if they would have to pick up the pieces.

### Picking up the pieces

This thematic finding attends to all five environments within the social ecological model and highlights the complexity of the new graduate nurse experience during the pandemic. This theme, *Picking Up the Pieces*, was the strongest and most salient theme in the data. Participants spoke to feeling they were left to pick up the pieces on multiple professional and personal levels. First, participants articulated picking up the pieces within themselves, as evidenced by having to rectify and reconcile what they thought nursing would be, to what it actually was, notably during a pandemic, with one participant telling us: “This is not how I thought I would start my nursing career.” A majority of participants also discussed having to work through the jarring moral distress experienced from enforcing Covid-19 visitor restriction policies which deeply challenged their ethical sensibilities regarding person and family centered care.

As a result of close proximity to patients and families, some new graduates remarked they were picking up the pieces of the emotional and mental recoil ensuing from the pandemic, noting high rates of psychological distress and mental health concerns “…now we’re dealing with a mental health pandemic.” This bearing witness of distress was emotionally taxing on participants, notably in the absence of a consolidative experience that may have allotted them the experiences of caring of patients and families experiencing psychological distress in a safe, supportive and closely mentored environment.

At a colleague level these new graduates were acutely aware of the immense burnout and psychological distress their more senior colleagues were experiencing. This actualized itself in practice in many ways; notably poor morale, with one participant describing their current work environment wherein, “it's [joy] been stolen, taken away,” with another participant noting, “there's a lot of trauma [notable in colleagues].” Another exacerbation of burnout and psychological distress was apathy towards mentorship, evidenced in both their own experiences of transition and the fact that some were now precepting 2021 new graduate nurses. There was an aura of fear in participants stories as it pertained to how they were to be the next generation of nurses as they witnessed such high rates of turnover and poor retention in their workplaces, with one participant noting “more staff are being hired but people are still leaving, they’re not retaining staff.” Another participant relayed their fear as follows:

“…knowing that so many senior nurses are leaving is scary, having new grads in areas where they won’t get that support [from senior nurses]…it's something a lot of us [new graduates] are thinking about…not having that mentorship and support, it is going to be very hard to build ourselves professionally if we don’t have that.”

Participants also felt they were witnessing a crumbling of the nursing profession, as evidenced by the public outcry from nurses during Covid-19 and the highly publicized lived realities of nursing in their first year of practice. One participant told us: “during the pandemic nurses were taken advantage of: underpaid, redeployed to places that were not safe.” Participants also felt the weight of the mass exodus of nurses in Canada, more specifically, the province of Ontario, noting that, for example: “The RNAO [Registered Nurses Association of Ontario] statistics on nurses leaving after the pandemic is terrifying.”

While witnessing the state of the profession they also felt the moral weight to reconstruct the nursing profession for themselves and future generations of nurses, articulating sentiments such as, “I think we have to rebuild the profession from the ground up after this [pandemic] is done” and, “The next five years are going to be really tough [for this nursing cohort].” They dealt with this all while contemplating what entering the profession during such upheaval meant for them. As one participant reflected, “I wonder what the repercussions of us entering the profession now are, feeling unprepared…I have another new graduate friend that is already burnt out.”

Lastly, at a systems level, all participants spoke about professional unrest in nursing, and interestingly, almost every participant brought up Bill 124 without prompt. Bill 124 is a wage cap legislation in Ontario that came into effect in 2021, capping nurses’ salary increases to one percent annually (Legislative Assembly of Ontario, 2021). Participants discussed at length how this particular bill and the current Ontario government made them feel worthless at a time when they expected nurses to be highly regarded and respected by government. Participants said things like, “it's really hard knowing the government doesn’t support you in the way they should compared to other professions, especially when we have been the most hard hit [by Covid]…it's demoralizing.” While another participant reflected on the gender discrimination they believed was behind the bill: “[The] government sees us as so little in comparison to firefighters and police officers…it feels like we [nurses] are fighting a battle on our own,” while another participant called on the government: “If you have so much respect for us, pay us what we’re worth,” this particular remark was in response to the Ontario premier repeatedly calling nurses heroes throughout the course of the pandemic while simultaneously legislating a wage cap for nurses salaries. These participants demonstrated a strong awareness of the intricate and complex relationships between government and nursing.

### Knowing when to say no and let go

This particular theme illuminates the complex, and dynamic ways in which the intrapersonal (individual characteristics) organizational (processes embedded in organizations) and community/sociocultural (values and beliefs of wider society) environments of the social ecological model shaped participant experiences. Participants clearly articulated their moments of success in the first year of transition and associated abilities to mitigate complex work environments to their previous life and career experiences. One participant reflecting on their experience, articulated:

“this would be so difficult coming out of my first degree – I would not have the insight I have now…to know that bad days are often the systems fault, not mine…but with my past life experience I can come to peace with that.”

As such, participants appeared to have a strong knowing of when to say no, and when to let go. Some spoke of knowing when to say no to work environments that contributed to burnout – for example, “I work with colleagues [in acute care] that are so burnt out and I’m not going to do that to myself.” Another participant reflected on their rationale for actively searching for a new workplace: “I wanted to be a nurse because I could be bigger and do better and I am getting that from nursing, but I am not getting that from my employer.” This participant, and others, were very clear in that they would not tolerate a work environment that did not enable them to be the best nurse they could be. Participants problematized the workplace, not the profession. It is worth noting that not one participant stated they were considering leaving the profession.

Participants also made it very clear that they did not buy into the historically constructed narratives that exist and surround the nursing profession. For example, a prominent sentiment was against the “nurse martyr” and “hero” complexes, which have once again come to the forefront of nursing discourse over the course of the pandemic. One participant pointedly cited the nurse martyr complex as “actively harmful.” Others also described overcoming other engrained narratives in the profession, for example, the notion of having to, “put in your time in acute care” before working in other areas of nursing. Half of these participants had already left acute care hospitals, and half of them did not even start their career in these environments. Those in acute care hospitals highlighted that acute care nursing was not a long-term career option as they said it fosters high levels of burnout alongside low levels of psychological safety. Participants consistently spoke to what they would do next in nursing. Many were already transitioning to community jobs that supported a better work life balance and offered more robust orientation programs. Multiple participants described a desire to quickly return to university to pursue graduate nursing programs, once again, letting go of the historically constructed belief that nurses have to work on the frontline for years before earning the right to attend graduate school.

Lastly, participants were very comfortable letting go of toxic work environments that did not support their transition needs or their own health and wellbeing, with one participant stating: “I’m too old for this crap…I can go [nurse] elsewhere…” while another reflected on their decision to leave their first nursing job:

“The way I saw it [nursing work] on the unit made me know this is not a safe or supportive place for new grads…I didn’t want to be in a position where I’m crying in a locker room begging for support and not receiving it…so I left during orientation.”

## Discussion

The findings from this study stimulate important considerations pertaining to the potential uniqueness of second entry new graduate nurse transition, the current state of the nursing workforce, and the concept of socio-political knowing – something that was exhibited by all participants. Each will be discussed below.

### Second entry new graduate nurses

New graduate nurses are rarely, if ever distinguished further than new graduate nurses – meaning those that enter the profession via alternative road maps, for example second entry or bridging (from a diploma to degree nursing program), are rarely identified as unique subsets of new graduate nurses. Second entry nursing graduates represent an unknown percentage of nursing graduates in Canada, as this data is currently not captured in publicly available statistics. This is problematic as the sparse body of literature on second entry new graduate nurse transition suggests they have some unique entry to practice experiences when compared to traditional new graduates, as noted in the background section of this paper and further substantiated by some of the findings from this study. For example, participants often said they felt they fared better than their traditional new graduate nurse colleagues. While these nursing professionals bring a wealth of experience from their previous careers, some research suggests they still share similar concerns and fears as they transition to independent practitioners (Cantlay et al., 2017; Shatto et al., 2016).

Traditional new graduate nurse transition experiences are said to be stressful and emotionally exhausting (Creswell & Plano, 2011) and often result in new graduate nurses feeling burnout and at times unsafe within their first year of practice (Parker et al., 2014). The findings presented in this study corroborates this for second entry new graduate nurses – there was still fear and unease in being a new graduate nurse, even though participants did clearly articulate they believed they fared better than others who were not second entry students. There remains a paucity of research examining the transition experiences of these nurses compared with their traditional counterparts (Rainbow & Steege, 2019), which is problematic as the data in this study does suggest their experiences have unique elements, even in the midst of a pandemic.

### State of the nursing workforce

Participants were clearly impacted by the current state of the nursing workforce, by way of both witnessing and experiencing. Participants witnessed immense burnout and a lack of psychological safety in their workplaces. This is supported in the literature, both during and predating Covid-19. In the context of the Covid-19 pandemic, psychological distress increased for nurses, as did moral distress, moral suffering, compassion fatigue, and burnout​ (Adams & Walls, 2020; Crowe et al., 2021; Duong, 2021; Hubert & Eichenberger, 2021; Lai et al., 2020; Manzano García & Ayala Calvo, 2021; Stelnicki et al., 2020; Stuijfzand et al., 2020). Attrition in nursing is also at an all-time high (Varner, 2021), which for participants meant very heavy workloads and insufficient mentorship. Both of these mediated participants understanding of, and fears about, their career trajectories. Participants not only discussed the urgent need to bolster mental health supports and address psychological safety in the workplace, but they also made career decisions based on these factors. Their calls for urgent action are reflected in recent research findings that highlight disproportionately high rates of burnout and mental health issues amongst nurses (Bourgeault et al., 2022). Furthermore, their calls for action at a systems level, and their understandings of the role gender plays in nursing workplace issues continues to be reflected in emerging health human resource literature (Ben Ahmed & Bourgeault, 2022; Bourgeault et al., 2022).

### Socio-political knowing

Socio-political knowing provides a conceptual way to understand some of the findings from this study. Socio-political knowing in nursing is understood as a type of knowing that “lifts the gaze of the nurse…and situates it within the broader context in which nursing and health care take place” (White, 1995, p. 83). It is the knowing that challenges nurses to question taken for granted assumptions about “practice, the profession and health policies” (White, 1995, p. 84). Socio-political knowing has two elements: the context of persons (patient, nurse, colleague, employer), and the context of the profession, which includes “both society's understandings of nursing and nursing's understanding of society and its politics” (White, 1995, p. 84). Based on this conceptualization of socio-political knowing, participants in this study exhibited prominent levels of engagement with the concept. For example, these participants set strong boundaries regarding what they will and will not tolerate before they leave a workplace, as evidenced in the theme ‘*Knowing When to Say No and Let Go*.’ Participants had exceptionally high levels of self-awareness and were able to clearly articulate their needs and boundaries. This was evidenced in the finding that half of the participants had already left their first nursing job, as it was not offering them the support they needed. Existing literature suggests that between 37–57% of new graduate nurses will leave their first nursing job by their second year of practice (Chandler, 2012; Laschinger et al., 2012), which was reflected in this study's findings. Interestingly enough, for this group of participants, the jobs they left tended to be acute care hospitals, in favor of nursing jobs outside of hospital settings.

Participants were also engaged in critical reflection regarding deeply engrained understandings of nursing, such as notions of self-sacrifice (noted by one participant as the “martyr complex”) and the hierarchical structures that influence the trajectory of nurses’ professional lives. Furthermore, they were acutely aware and critical of the ways in which government influenced, and negatively impacted nurses and their work, which they linked to poor mental health outcomes for nurses. They further engaged with socio-political agency by clearly articulating how they have integrated their reflections into decisions about their planned career trajectories.

This study has suggested that for multiple reasons, key interventions that support new graduate nurse transition and retention were not met, including strong mentorship, protected orientation time, and psychological safety in the workplace, alongside robust mental health supports. These are not new concerns, but the pandemic has amplified them at a time when the profession needs new graduate retention more than ever. The experiences of new graduate nurses during a global pandemic provides important insight into how competing priorities during a health crisis must be more carefully evaluated and weighed by decision makers – it is evident in the findings from this study that resource scarcity and further redirection to Covid-19 responses impacted transitional supports for these new graduate nurses. Recommendations regarding new graduate nurse transition as they relate to the Covid-19 pandemic are described below.

Formal and informal mentorship is necessary, during and beyond the pandemic. Orientation programs are the foundation for success for new graduate nurses (Hussein et al., 2017). Fulsome orientation is a strongly established contributor to new graduate nurse retention (Rohatinsky et al., 2017) however, it is not consistently incorporated into new graduate onboarding processes. Even predating the Covid-19 pandemic, mentoring supports fall short for many reasons, most notable chronic staffing shortages (Twibell et al., 2012). In the context of the Covid-19 pandemic, resource re-allocation to Covid-19 responses has been noted to affect other areas of funding for nurses, which may have also impacted the availability of resources for new graduate nurses in various healthcare settings. For example, it has been recommended that additional training resources be given to new graduate nurses entering the profession during Covid-19, in light of the significant loss of clinical hours due to clinical agencies restricting student access during the pandemic (ONL, 2021). However, additional transition resources and supports were not provided to participants in this study, which exacerbated their levels of stress and feelings of inadequacy. Protected orientation time is necessary, yet participant orientation in this study was said to be cut short as a response to exorbitant staffing shortages. Orientation should not be cut short, however, in practice this continues to occur, having significant implications on new graduate nurses – both decreasing their confidence as an independent practitioner and increasing their desire to leave the workplace (Kiel, 2020; Lindfors et al., 2022; Schmidt & Schiffman, 2019).

This study stimulates new research questions regarding our understandings of new graduate nurse transition. For example, what are the differences between second entry and traditional new graduate nurses as it relates to navigating transition into practice? Secondly, what differences, if any, exist between second entry and traditional new graduate nurses understanding of, and engagement with the socio-political contexts of the profession? And how may this shape their experiences and mediate their intent to stay or leave, and more broadly, influence their nursing identity? It is also imperative to reflect upon and seek to clarify the roles and responsibilities of educational institutions and employers to address gaps in knowing and doing in new graduate nurses, particularly as it pertains to unforeseen circumstances like a global health crisis.

### Study limitations

A sample size of eight serves as a limitation in that findings are not generalizable. Another limitation is that all participants were practicing in only one province in Canada. Future research should seek to explore second entry new graduate nurse transition experiences nationally and internationally.

## Conclusion

In sum, this study reports relevant and timely findings and raises important questions about the variance in new graduate nurse experiences and the significance of engaging with, and the enactment of socio-political practice in early career stages. Multiple waves of Covid-19 are ongoing globally, and pandemics will continue to occur with increasing frequency (Monto & Fukuda, 2019; Ross et al., 2015; WHO, 2020), creating situations that will again require fast-evolving healthcare responses which impact the work environments of new graduate nurses. It is therefore essential to understand all facets of new graduate nurses’ experiences of transition during Covid-19, including those graduates that do not follow tradition trajectories of obtaining a nursing degree. Learning from these non-traditional graduates can aid in ensuring appropriate supports are put in place for healthy transition and ongoing retention of *all* new graduate nurses. It is equally important that pandemic specific policy development regarding new graduate nurse transition be based on emerging evidence.
